# Rheological Characterization as an Alternative Method to Indentation for Determining the Setting Time of Restorative and Endodontic Cements

**DOI:** 10.3390/ma10121451

**Published:** 2017-12-20

**Authors:** William N. Ha, Timothy M. Nicholson, Bill Kahler, Laurence J. Walsh

**Affiliations:** 1 School of Dentistry, University of Queensland, Herston, QLD 4006, Australia; wyattkahler@bigpond.com (B.K.); l.walsh@uq.edu.au (L.J.W.); 2 School of Chemical Engineering, University of Queensland, St. Lucia, QLD 4072, Australia; t.m.nicholson@uq.edu.au

**Keywords:** mineral trioxide aggregate, elastic modulus, dental materials, glass ionomer cement, epoxy resin cement, glass ionomer, Biodentine, bioceramics, endodontics, physical properties

## Abstract

This study explored an alternative approach using rheology to assess setting time. The following cements were tested: ProRoot^®^ MTA (Dentsply, Tulsa, OK, USA), Biodentine^®^ (Septodont, Saint Maur des Fosses, France), Fuji VII^®^, FujiVII^®^ EP, and Fuji IX^®^ (from GC Corporation, Tokyo, Japan), RealSeal SE™ Sealer (SybronEndo, Amersfoort, The Netherlands), AH 26^®^ and AH Plus (both from Dentsply DeTrey, Konstanz, Germany). Freshly mixed cements were placed into a strain-controlled rheometer (1 rad·s^−1^ with an applied strain of 0.01%). From measurements of elastic modulus over time, the time taken to reach 90% of the plateau elastic modulus (designated as the setting time) was determined for each cement. In increasing order, the setting times were as follows: Fuji VII EP 3.3 min, Fuji VII 3.6 min, Fuji IX 3.7 min, ProRoot MTA 5.1 min, Biodentine 15.9 min, RealSeal 22.2 min, AH Plus 5933 min, and AH 26 5067 min. However, ProRoot MTA did not yield reliable results. The time to reach the 90% plateau elastic modulus correlates well with the setting time of glass ionomer cements and Biodentine. Using this approach gives much longer setting times for endodontic sealers than previously recognized.

## 1. Introduction

The Academy of Prosthodontics defines dental cements as “binding elements or agents used to make objects adhere to each other, or something serving to firmly unite”, i.e., a luting agent [1], while the international standard for dental vocabulary defines dental cements as materials for “luting of dental prostheses and lining or base filling of prepared teeth, or, the substitution of missing parts of teeth” [2]. Furthermore, the American Association of Endodontists defines a “root canal sealer” as “a radiopaque dental cement used, usually in combination with a solid or semi-solid core material, to fill voids and to seal root canals during obturation; included are bioceramics, resins, calcium hydroxide, zinc oxide-eugenol, glass ionomers (GICs) and others” [3].

There does not appear to be any formally accepted definition for dental cements that encompasses luting agents, liners, bases, restorative cements and endodontic sealers, although from a clinical end-user perspective they all could be grouped under a definition such as “any material that requires mixing of components resulting in a hardening colloid that progressively sets to become a solid.”

Of the two commonly referenced testing methods for testing dental cements, ISO 9917-1 details methods for testing the properties of zinc phosphate, zinc polycarboxylate, and GICs, while ISO 9917-2 describes the same approach for resin-modified cements and ISO 6876 details methods for testing the properties of endodontic sealing materials [4,5]. The method used to assess setting time in both sets of standards involves placing a weighted circular needle (Gillmore needle) perpendicularly onto the setting cement. If the needle leaves a circular indentation, the cement is deemed to be unset. If the needle does not leave a circular indentation, then the cement is deemed to be set. In ISO 9917-1, the Gillmore needle has a mass of 0.4 kg and a diameter of 1 mm and applies a pressure of 4.99 MPa [5]. In ISO 6876, the Gillmore needle has a mass of only 0.1 kg, a diameter of 2 mm and so applies a pressure of only 0.312 MPa [4]. The logic that underpins the 16-fold difference in the pressures applied between the two standards appears to be that restorative cements are expected to sustain greater loading under function.

A key issue when interpreting indentation setting times is that the outcomes at any one point in time are dichotomous since the material is defined as either ‘unset’ or ‘set’. The indentation approach gives no insight into the progression of the setting reaction. It is possible that the definition of ‘set’ could erroneously be interpreted as meaning ‘completely reacted’ when in fact the setting reactions of the cement have not yet reached completion [6].

A different approach to determining the setting time is described in ISO 4049 for resin dental materials and specifically refers to changes in temperature over time that reflect the exothermic nature of the setting reaction which, when graphed, resembles a sigmoid curve. Setting time is determined by extending a horizontal line from the plateau to meet an extension of the straight line along the temperature increase [7]. This point of intersection occurs just before the temperature reaches a plateau.

**Figure 1:**
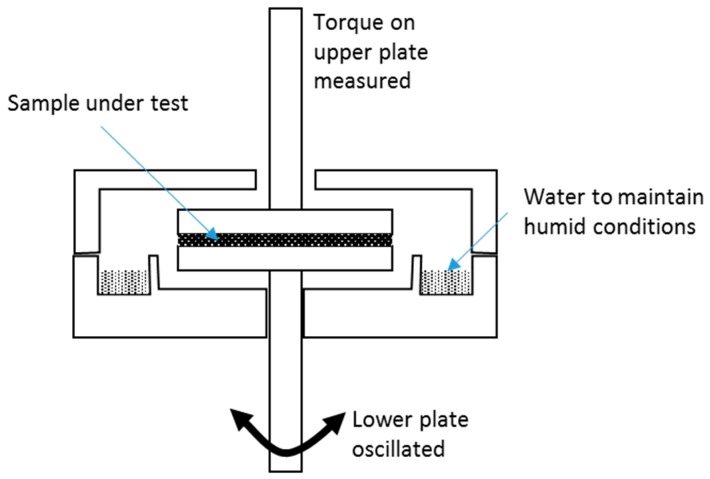
Parallel plates in a rheometer. A chamber can be constructed to maintain humidity and prevent desiccation of samples.

A third approach to measuring setting times is to track changes in the rheological properties of the materials over time using small strain oscillatory deformations. In this technique, the material is placed between two surfaces and a small amplitude sinusoidal shear strain deformation applied. By measuring both the amplitude and relative phase of the resulting stress response the rheological properties of the material can be determined. For many complex materials, such as the hydrocolloids being considered here, this response is termed ‘viscoelastic’ as the material has both elastic (solid-like, the energy stored in the deformation cycle) and viscous (liquid-like, the energy dissipated in the deformation cycle) components. The material response is therefore characterized by both an elastic modulus (G’) and a viscous modulus (G”). A small strain is used so as to limit any changes in the microstructure of the system during the test. In practice, the geometry most often used is either parallel plates or a cone and plate, which are rotated about their common axis (Figure 1). Further details of these rheological techniques can be found in standard texts (for instance Macosko 1994 [8]).

For the purposes of measuring the setting of a dental cement, initially the material will exhibit viscoelastic behaviour with some liquid-like properties, but as the material sets, it will become an elastic solid. Therefore, the elastic modulus is an appropriate measurement to consider when considering setting reactions. Tracking the G’ over time can document the progression of a mixture from a liquid-like state (with less resistance) to a solid-like state (with greater resistance) [6]. As time progresses and the material sets the G’ will asymptotically approach a plateau value. One could then set a threshold such as 90 or 95% of the final modulus to define an endpoint [6], although the selection between such specific threshold values is arbitrary. Logically, the definition of when the material sets should correspond closely with its clinical manipulation.

Rheological properties of endodontic sealers have been assessed in prior studies. Volumetric flow rate, as measured using a capillary rheometer, has been used to measure the flow properties of endodontic sealers [9] as an alternative to assessing film thickness, as described in ISO 6876 [4]. This approach measures the velocity and volume of sealer as it enters capillary tubes at different strain rates. While this method is not useful for measuring setting time, its use does highlight the applicability of rheometric analyses of the flow properties of endodontic sealers. Endodontic sealers exhibit viscoelastic properties [10], with the viscosity affected by the applied shear rate [11]. These materials exhibit a shear-thinning behaviour (i.e., the apparent viscosity decreases with increasing shear rate), particularly as the powder content rises and the viscosity of the freshly mixed cement increases. 

The setting reaction of glass ionomer cement (GIC) involves aluminosilicate glass particles reacting with polycarboxylic acid, producing a gel within which polyacid chains cross-link to produce the set material [12]. Under ISO 9917-1, GIC luting agents are expected to set under 8 min, while GIC used as bases, liners and bulk restoratives are expected to set under 6 min. The setting reaction of GIC continues long after the indentation-designated setting time and is described as ‘maturation’ [13].

Rheometry has also been used to explore the setting reactions of GICs and has been used to determine setting time [14,15,16]. These cements show a progressive increase in viscosity over time, as the material sets. However, past work on this topic did not propose a definition of setting time based on viscosity. Rather, Algera proposed the setting time as being the point in time when the threshold of 95% of the material’s maximum displacement was reached [14]. A difficulty with this approach is that in a material that exhibits viscoelastic properties, displacement will be affected by the rate that the stress is applied. As a consequence, the value of displacement is relevant only to the specific experimental settings used.

Rheometry has also been used to explore the setting reactions of mineral trioxide aggregate (MTA) cements. MTA is typically a mixture of 80% Portland cement with 20% bismuth oxide [17]. The principal setting reactions occur between tricalcium silicate, dicalcium silicate and water, producing various calcium silicate hydrates [17]. Under ISO 9917-1, which is named “water-based cements”, the setting time defined using an indentation test is 4 h. This value does not align with its clinical use since in most cases MTA is overlaid with a permanent material within the same appointment [6,18,19,20]. A similar situation exists for Biodentine (Septodont, Saint-Maur-des-Fossés, France), a material that has setting reactions that are broadly similar to MTA but with a shorter setting time [21]. The changes in viscosity of an experimental MTA cement have been assessed using a strain-controlled rheometer with two parallel plates to measure the changes in elastic modulus (G’) [6]. This provides support for the concept of using rheological properties to determine the setting time of MTA cements.

The aim of the present study was to assess changes in G’ over time for a range of dental materials (MTA), Biodentine, two GICs, two epoxy resin sealers (AH26 and AH Plus) and a methacrylate resin sealer (RealSeal SE). Setting time was defined as the time to reach 90% of the plateau G’. AH 26^®^ (Dentsply DeTrey, Konstanz, Germany) is an epoxy resin cement supplied as a powder that is mixed with a liquid (bisphenol diglycidyl ether). The powder contains bismuth oxide (as the radiopaque agent) and hexamethylenetetramine (HMT), which serves as the hardener. When mixed, the HMT crosslinks the bisphenol diglycidyl ether to produce a three-dimensional network, hence making the material set into a hard mass [22]. A related epoxy resin cement is AH Plus^®^ (Dentsply DeTrey, Konstanz, Germany), which is supplied as two pastes that are mixed together, with silicon oil as a carrier. The epoxy resin component is diepoxide, while the hardener component is a polyfunctional amine mixed with silicon oil. Both cements components also contain fumed silica as an inert thickener [23]. RealSeal SE™ (SybronEndo, Amersfoort, Netherlands) is a fourth-generation methacrylate resin sealer based on 4-methacryloxyethyl trimellitic anhydride, a monomer that polymerizes with tri-n-butylborane [24].

## 2. Materials and Methods

### 2.1. Sample Preparation

ProRoot^®^ MTA powder (Dentsply, Tulsa, OK, USA, lot No. 13102907) was mixed with distilled water to a ratio of 0.40 by weight. This mixing ratio was-based upon a compromise between the water:powder ratio of 0.33, as recommended in the patent [17] and the ideal water:powder ratio of 0.42 required when mixing water with Portland cement to achieve complete hydration without supplying extra water [25]. This ratio of 0.40 for mixing water with MTA powder has been used by other studies [26,27]. The powder and water mix was placed into a capsule that was agitated in an amalgamator for 30 s at 4600 oscillations per minute. The use of a capsule to mix the cement aligns with previous studies [6,28]. In a similar manner, Biodentine powder (lot No. B05594) in capsules was mixed with the provided liquid in an amalgamator for 30 s at 4600 oscillations per minute.

AH 26 (lot No. 1406001107) was mixed by combining 2 volumes of powder with 1 volume of resin (paste), while AH Plus (lot No. 1406000958) was prepared from the two component pastes mixed at equal weights. Likewise, RealSeal SE (lot No. 4971883) was prepared using the supplied automatic mixing tips.

Fuji VII^®^ (GC Corporation, Tokyo, Japan, lot No. 1303041), Fuji VII^®^ EP (lot No. 1108031) and Fuji IX^®^ GP Extra (GC Corporation, Tokyo, Japan, lot No. 1111261) were supplied in capsules that were mixed in an amalgamator for 10 s at 4600 oscillations per minute.

All mixed samples were immediately placed into the rheometer for testing.

### 2.2. Rheological Testing

The measurement of elastic modulus (G’), for various dental cements during the setting process followed the protocol used in a previous study [6]. In brief, testing was performed using an ARES strain-controlled rheometer (Advanced Rheometric Expansion System, TA Instruments, New Castle, DE, USA), within which freshly mixed cements were placed between two parallel 7.9 mm diameter circular plates separated by a 0.6–0.7 mm gap. These minor variations in the gap distance between the plates were the result of some samples exhibiting higher resistance to compression. However, the differences in gap distance are incorporated in the calculations for G’, so are not a confounding factor.

An enclosed chamber was used to maintain constant conditions for both temperature and humidity (Figure 1). The lower plate was maintained at a constant temperature of 38 °C (±1 °C). The relative humidity was kept at 100% by a trough containing water that was included in the chamber. The rheometer conditions were an applied strain of 0.02% with a 1 radians per second oscillation frequency. These conditions apply less strain than would alter the structure of the material, approximately 0.05%, a threshold established by performing a strain amplitude sweep on the cements where, at low amplitudes, the G’ and G” are approximately linear (i.e., amplitude independent) then falling when the critical strain is reached. Thus, the critical strain is obtained from plotting a log-log graph by extrapolating the linear portion and the decreasing portion of the curve.

Each material was tested in triplicate.

## 3. Results

**Figure 2:**
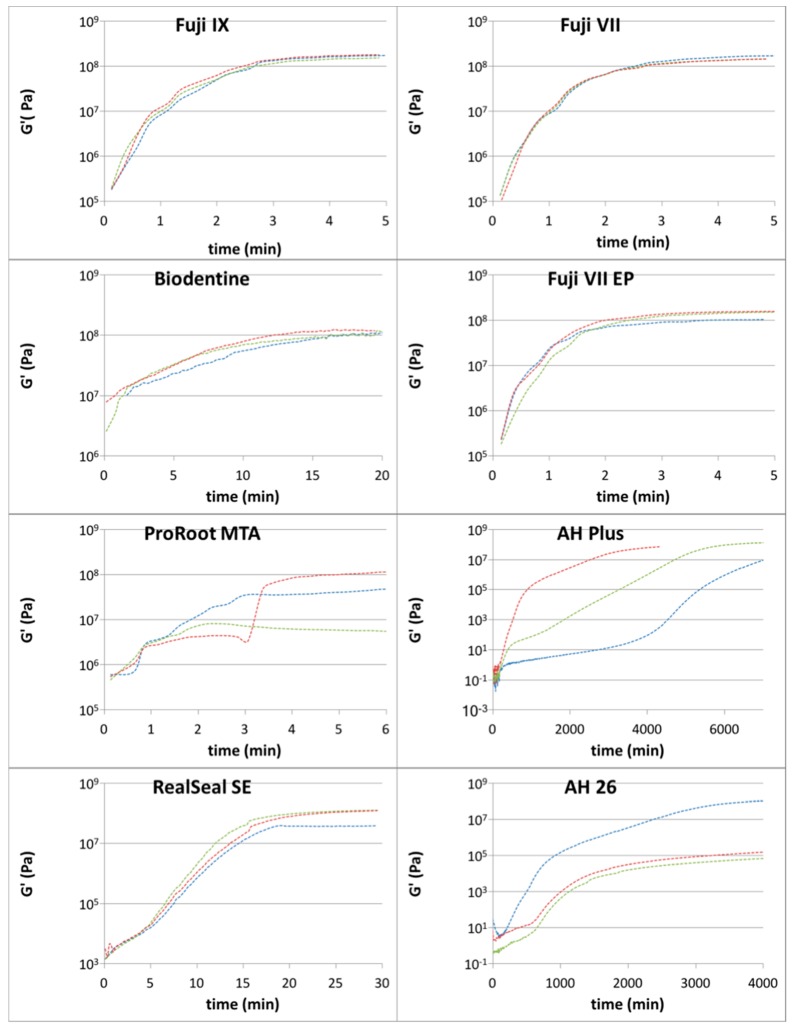
Elastic (G’) modulus of tested dental cements over time. Different color plots are the trend lines for different replicate samples of the same material.

Representative rheological data are presented in Figure 2, while data for plateau G’ (in MPa) are given in Table 1. The calculated setting times based on the time to reach 90% of the plateau G’ are given in Table 2. 

In increasing order, the setting times were as follows: Fuji VII EP: 3.3 min, Fuji VII: 3.6 min, Fuji IX GP Extra: 3.7 min, Biodentine: 15.9 min, RealSeal SE 22.2 min. These values illustrated margins of error below 5%. However, the remaining setting times of AH Plus: 5933 min, AH 26: 5067 min and ProRoot MTA: 5.1 showed larger margins of error. 

A comparison of marketed setting times against proposed setting times using the time to reach 90% of the plateau elastic modulus is given in Table 3.

## 4. Discussion

The current common definitions for dental cements are based on their clinical uses rather than their chemical properties. As many materials can have multiple clinical uses, the term “dental cement” should be defined by the setting properties as primarily chemical in nature and becomes progressively more rigid over time.

The ISO 9917-1 standard “Dentistry—water-based cements” specifies methods for testing the properties of zinc phosphate, zinc polycarboxylate, and glass polyalkenoate cements [5]. It does not include in its scope MTA or any ‘bioceramics’. The major differences between the materials listed in this ISO standard and MTA or ‘bioceramics’ are that the materials specified in the standard react with aqueous fluids of various types containing components such as phosphoric acid or polyacrylic acid, while with MTA and bioceramics, the powder reacts with pure water. ISO 9917-1 assumes that the setting reaction has completed after one hour and at this point in time the material is ready for compressive strength testing. Because the ISO standard does not consider ‘wet curing’, testing of MTA and bioceramics can yield unexpected or inferior results [28].

The use of separate testing standards for restorative materials and endodontic sealers creates confusion when interpreting the literature on materials used for root repair. For example, ProRoot MTA, when tested using indentation as per ISO 6876 showed a setting time of 78 min, but when tested using indentation as per ISO 9917-1 showed a setting time of 261 min [19]. As the clinical indications of root repair materials overlap with general restorative materials and endodontic sealers, so will their properties. Therefore, applying a single methodology for testing all three categories of materials, as was done in this study, can provide direct and meaningful comparisons of materials. 

In the present study, the sample size was 3, as this is the requirement of both ISO 9917-1 and ISO 6876. These standards provide no guidance on how variations between replicate samples are described, whether it be as upper and lower limits, standard deviations or standard errors. Likewise, there is no consistent approach in the literature on dental cements. For example, utilizing the ISO 6876 indentation test, AH Plus has reported setting times of 960 min (SD 79 min) [10], and 690 min (SD 90 min) [29]. Using the same indentation test for Biodentine has produced setting times of 86 min (SD 6 min) [30] and 45 min [31]. With indentation tests, the interpretation of when complete indentation fails to occur is subjective. Therefore, despite using more replicate samples, indentation testing will not give improved accuracy or reproducibility, as there will be variations due to the testing operator. In contrast, the use of calculated measurements from testing using a rheometer should give better reproducibility across different testing laboratories.

Regardless of the methods being used, manufacturers should disclose the extent of variations that occur in setting time, including both range and SD. This would enable clinicians to better understand the anticipated clinical handling of the product being used.

The present results for endodontic sealers show values for the rheological setting time that are substantially longer than those that are advertised. Such differences may have implications for clinical practice, for example when sealer that has not yet fully set could be displaced, compromising the seal. This could occur if post space preparation is performed soon after obturation of the root canal. 

In the present study, a decision was taken to use 90% of the plateau elastic modulus as setting time, rather than 95% as had been used previously [6], The major reason for this is that the clinical experience of the authors has shown that the materials used in the study can be easily displaced at their marketed setting time. The time to reach 90% of the plateau elastic modulus appears to represent a clinically useful point for how restorative cements as well endodontic sealers behave in the clinical environment. This value provides a single test definition for all types of materials.

In the present study, ProRoot MTA was mixed in a method similar to that used with Biodentine. One sample showed progressive increases in elastic modulus, to give a rheological setting time of 7 min. Compared to the marketed setting time of 4 h, the time period of 7 min is far closer to the time after mixing when the material can be overlaid in the clinical setting. As an example, overlaying MTA with bonding resin 10 min after mixing has been proposed by Tsujimoto [18]. Likewise, placing GIC over MTA after 45 min has been suggested by Yeilyurt [32]. However, as seen in Figure 2, samples of ProRoot MTA did not show the same pattern of gradual increase in elastic modulus over time as seen in other materials. In one sample, there was a discontinuity in the trend curve, which was likely due to loss of structural integrity of the sample. This could be explained by the nature of highly concentrated and non-homogenous suspensions of solid particles in a liquid [33]. Therefore, responses to stress during the setting process are inherently variable.

The results of the present study show that curves showing the progression of G’ vary between materials. The GIC samples had almost identical curves, with a steep gradient. The setting reaction of GIC involves progressive cross-linking of polycarboxylic acid chains by Ca^2+^ forming a homogenous polymer network [16]. Mixing the GIC in an amalgamator ensures consistent interactions between the powder and liquid components. Biodentine samples gave curves with a lower gradient, representing a slower setting reaction as tricalcium silicates react with water [34]. This is in contrast to ProRoot MTA, where both calcium silicates and calcium aluminates react with water to creating a matrix [35]. The setting reaction of AH 26 relies on the slow polymerization of epoxy resin with bisphenols [22]. In contrast, AH Plus, a chemically related material, shows a lower initial gradient. One explanation is that AH Plus has components dispersed into silicon oil, which is an inert carrier, while AH 26 is a power mixed into a liquid without any carriers being present.

Factors related to material handling can also contribute to variability in the results seen. For ProRoot MTA, the instructions for use do not specify a particular water-to-powder ratio but instead suggest adding water to the powder until a thick creamy consistency is achieved. It must, therefore, be stated that the setting time of ProRoot MTA in clinical practice will vary according to the water-to-powder ratio chosen by the clinical end user.

The use of capsules with pre-dispensed amounts of powder and liquid eliminates variations in setting time due to inherent variations in dispensing. The dispensing of proportions is imprecise, the time spent handmixing will vary between samples, and the integration of the components from mixing will vary between samples. In the present study, materials which were dispensed and mixed by hand (ProRoot MTA, AH Plus, AH 26) displayed greater variation in their setting properties than those dispensed in capsules (Biodentine, Fuji VII, Fuji VII EP and Fuji IX) or automatically mixed (RealSeal SE). Taking this into account, manufacturers of hand-mixed materials should state the setting time as a range, rather than as a precise value.

Changes in temperature can be used to determine how a setting reaction is progressing, and this parameter is used for following polymerization of dental resin cements [7]. Following a similar principle, the heat of hydration using isothermal calorimetry could be applied to dental cements [36]. However, rheological studies can measure the elastic modulus and therefore measure both the working time and the setting time within a single test. However, further research is required to determine which points in the elastic modulus curve could best be defined as the working time [10].

The rheometric method of measuring setting time provides several advantages over the traditional method of indentation testing. Indentation testing relies upon human judgment to determine whether or not a material has set, while the rheological method is objective and does not require human judgment. The use of a closed chamber enables control of humidity and temperature and removes variability due to oxygen inhibition of resin polymerization, or desiccation of water-based cements during setting. While the testing environment of the rheometer is closer to clinical conditions in terms of humidity and temperature than testing on the bench, it cannot replicate all clinical variables that could potentially influence setting reactions when a material is placed into the tooth. Such factors include the presence of other fluids such as endodontic irrigants, dentinal fluid, and blood, which influence the setting reaction of MTA [37].

## 5. Conclusions

The time to reach the 90% plateau elastic modulus represents the elastic modulus is approaching an asymptote of the ultimate elastic modulus. This single method can be applied to multiple types of dental cements, and hence can be a uniform means for providing meaningful setting times for clinicians. It should replace the range of setting time tests that are used currently for different dental cements. The use of a rheometer can provide objective and reproducible results, which overcome problems of subjective interpretation, which affect indentation tests. Because materials that are hand-mixed will have greater variability, manufacturers should provide a range of setting times to reflect the variability that may occur under clinical conditions.

## Figures and Tables

**Table 1:** Plateau elastic modulus (in MPa).

	n	Mean (min)	SD (min)	SE	Margin of Error ^1^ (min)	Upper Bound (min)	Lower Bound (min)	Max (min)	Min (min)	Range (min)
Fuji IX GP Extra	3	1.7 × 10^8^	1.5 × 10^7^	8.8 × 10^6^	1.73 × 10^7^	1.84 × 10^8^	1.49 × 10^8^	1.80 × 10^8^	1.50 × 10^8^	3.00 × 10^7^
Fuji VII	3	1.5 × 10^8^	1.7 × 10^7^	1.0 × 10^7^	1.96 × 10^7^	1.70 × 10^8^	1.30 × 10^8^	1.70 × 10^8^	1.40 × 10^8^	3.00 × 10^7^
Fuji VII EP	3	1.4 × 10^8^	3.2 × 10^7^	1.9 × 10^7^	3.64 × 10^7^	1.73 × 10^8^	1.00 × 10^8^	1.60 × 10^8^	1.00 × 10^8^	6.00 × 10^7^
Biodentine	3	1.1 × 10^8^	5.8 × 10^6^	3.3 × 10^6^	6.53 × 10^6^	1.20 × 10^8^	1.07 × 10^5^	1.20 × 10^8^	1.10 × 10^8^	1.00 × 10^7^
ProRoot MTA	3	6.4 × 10^7^	5.6 × 10^7^	3.2 × 10^7^	6.33 × 10^7^	1.27 × 10^8^	7.20 × 10^8^	1.20 × 10^8^	8.10 × 10^8^	1.12 × 10^8^
RealSeal SE	3	9.6 × 10^7^	5.0 × 10^7^	2.9 × 10^7^	5.65 × 10^7^	1.53 × 10^8^	3.99 × 10^7^	1.30 × 10^8^	3.90 × 10^7^	9.10 × 10^7^
AH 26	3	4.3 × 10^7^	7.5 × 10^7^	4.3 × 10^7^	8.48 × 10^7^	1.28 × 10^8^	−4.13 × 10^7^	1.30 × 10^8^	1.80 × 10^5^	1.30 × 10^8^
AH Plus	3	7.5 × 10^7^	6.4 × 10^7^	3.7 × 10^7^	7.19 × 10^7^	1.47 × 10^8^	2.72 × 10^6^	1.40 × 10^8^	1.30 × 10^7^	1.27 × 10^8^

^1^ Confidence coefficient is set at 1.96. SD: Standard deviation; SE: Standard error.

**Table 2:** Time to reach 90% of the plateau elastic modulus (in minutes).

	n	Mean (min)	SD (min)	SE	Margin of Error ^1^ (min)	Upper Bound (min)	Lower Bound (min)	Max (min)	Min (min)	Range (min)
Fuji IX GP Extra	3	3.7	0.1	0.0	0.1	3.8	3.7	3.8	3.7	0.1
Fuji VII	3	3.6	0.2	0.1	0.2	3.8	3.4	3.8	3.5	0.3
Fuji VII EP	3	3.3	0.3	0.2	0.3	3.6	3.0	3.5	3.0	0.5
Biodentine	3	15.9	2.8	1.6	3.1	19.0	12.7	18.5	13.0	5.5
ProRoot MTA	3	5.1	2.8	1.6	3.2	8.3	1.9	7.6	2.0	5.6
RealSeal SE	3	22.2	3.6	2.1	4.1	26.3	18.2	24.4	18.1	6.3
AH 26	3	5066.7	1674.3	966.7	1894.7	6961.3	3172.0	7000.0	4100.0	2900.0
AH Plus	3	5933.3	1686.2	973.5	1908.1	7841.5	4025.2	7100.0	4000.0	3100.0

^1^ Confidence coefficient is set at 1.96. SD: Standard deviation; SE: Standard error.

**Table 3:** Comparison of marketed setting times against proposed setting times using the time to reach 90% of the plateau elastic modulus.

Dental Materials	Clinical Usage	Average Time to 90% Plateau G’ (min)	Marketed Setting Time ^1^ (min)	Testing Method of Setting Time ^1^
Fuji IX GP Extra	General restorative	3.7	2.0	ISO 9917-1
Fuji VII	General restorative	3.6	2.5	ISO 9917-1
Fuji VII EP	General restorative	3.3	2.5	ISO 9917-1
Biodentine	Root repair	15.9	12.0	Not stated
ProRoot MTA	Root repair	5.1	240	Not stated
RealSeal SE	Endodontic sealer	22.2	45.0	ISO 6876
AH 26	Endodontic sealer	5933	540 to 900	ISO 6876
AH Plus	Endodontic sealer	5066	Minimum 480	ISO 6876

^1^ According to manufacturer’s product information.
